# Rapid stromal remodeling by short‐term VEGFR2 inhibition increases chemotherapy delivery in esophagogastric adenocarcinoma

**DOI:** 10.1002/1878-0261.12599

**Published:** 2020-03-03

**Authors:** Anne Steins, Remy Klaassen, Igor Jacobs, Matthias C. Schabel, Monique G. J. T. B. van Lier, Eva A. Ebbing, Stefanie J. Hectors, Sander W. Tas, Chrissta X. Maracle, Cornelis J. A. Punt, Maria Siebes, Jacques J. G. H. M. Bergman, Jan Paul Medema, Johanna W. Wilmink, Ron A. A. Mathot, Gustav J. Strijkers, Maarten F. Bijlsma, Hanneke W. M. van Laarhoven

**Affiliations:** ^1^ Laboratory for Experimental Oncology and Radiobiology Center for Experimental and Molecular Medicine Cancer Center Amsterdam Amsterdam UMC University of Amsterdam The Netherlands; ^2^ Department of Medical Oncology Amsterdam UMC University of Amsterdam The Netherlands; ^3^ Oncode Institute Amsterdam UMC University of Amsterdam The Netherlands; ^4^ Department of Biomedical Engineering Biomedical NMR Eindhoven The Netherlands; ^5^ Oncology Solutions Philips Research Eindhoven The Netherlands; ^6^ Advanced Imaging Research Center Oregon Health and Science University Portland OR USA; ^7^ Department of Biomedical Engineering and Physics Amsterdam UMC University of Amsterdam The Netherlands; ^8^ Translational and Molecular Imaging Institute Icahn School of Medicine at Mount Sinai New York NY USA; ^9^ Department of Rheumatology and Immunology Amsterdam UMC University of Amsterdam The Netherlands; ^10^ Department of Experimental Immunology Amsterdam UMC University of Amsterdam The Netherlands; ^11^ Department of Gastroenterology and Hepatology Amsterdam UMC University of Amsterdam The Netherlands; ^12^ Department of Hospital Pharmacy Amsterdam UMC University of Amsterdam The Netherlands

**Keywords:** anti‐angiogenic therapy, chemotherapy, DCE‐MRI, esophagogastric adenocarcinoma, stromal remodeling

## Abstract

Anti‐angiogenic agents combined with chemotherapy is an important strategy for the treatment of solid tumors. However, survival benefit is limited, urging the improvement of combination therapies. We aimed to clarify the effects of vascular endothelial growth factor receptor 2 (VEGFR2) targeting on hemodynamic function and penetration of drugs in esophagogastric adenocarcinoma (EAC). Patient‐derived xenograft (PDX) models of EAC were subjected to long‐term and short‐term treatment with anti‐VEGFR2 therapy followed by chemotherapy injection or multi‐agent dynamic contrast‐enhanced (DCE‐) MRI and vascular casting. Long‐term anti‐VEGFR2‐treated tumors showed a relatively lower flow and vessel density resulting in reduced chemotherapy uptake. On the contrary, short‐term VEGFR2 targeting resulted in relatively higher flow, rapid vasodilation, and improved chemotherapy delivery. Assessment of the extracellular matrix (ECM) revealed that short‐term anti‐angiogenic treatment drastically remodels the tumor stroma by inducing nitric oxide synthesis and hyaluronan degradation, thereby dilating the vasculature and improving intratumoral chemotherapy delivery. These previously unrecognized beneficial effects could not be maintained by long‐term VEGFR2 inhibition. As the identified mechanisms are targetable, they offer direct options to enhance the treatment efficacy of anti‐angiogenic therapy combined with chemotherapy in EAC patients.

## Graphical abstract

Anti‐angiogenic therapies are combined with chemotherapy to treat cancers. Here, we reveal that therapy efficacy is greatly improved through previously unrecognized effects of VEGFR2 inhibition on tumor stroma in esophagogastric adenocarcinoma. Short‐term VEGFR2 inhibition results in activated fibroblasts that express FGF2 and hyaluronidase‐2, which enhances NO synthesis and hyaluronan degradation. This results in vasodilation, improved intratumoral perfusion, and chemotherapy delivery.

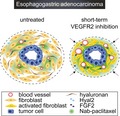

**Abbreviations**
CAF: cancer‐associated fibroblast
DCE: dynamic contrast‐enhanced
DWI: diffusion‐weighted imaging
EAC: esophagogastric adenocarcinoma
ECM: extracellular matrix
FGF2: fibroblast growth factor 2
HA: hyaluronan
HYAL2: hyaluronidase‐2
MRI: magnetic resonance imaging
NO: nitric oxide
NOS3: nitric oxide synthase 3
PDX: patient‐derived xenograft
TGF‐b: transforming growth factor beta
TME: tumor microenvironment
VEGFR2: vascular endothelial growth factor receptor 2
α‐SMA: alpha‐smooth muscle actin

## 1 Introduction

Angiogenesis is important for the growth of the majority of tumor types, and therapies directed against vascular endothelial growth factor (VEGF) and its receptor VEGFR2, combined with cytotoxic agents, have been used successfully to inhibit tumor growth in various solid cancers (Hurwitz *et al.*, 2004; Miller *et al.*, 2007; Sandler *et al.*, 2006). Ramucirumab, a humanized monoclonal antibody against VEGFR2, combined with paclitaxel has been approved by the US Food and Drug Administration (FDA) for the treatment of advanced esophagogastric adenocarcinoma (EAC) (Fuchs *et al.*, 2014; Wilke *et al.*, 2014; Young *et al.*, 2015). Although overall survival of patients receiving paclitaxel plus ramucirumab is significantly prolonged compared to paclitaxel monotherapy, the absolute benefit remains small (median 9.6 months vs. 7.4 months), urging the need to improve this combination therapy (Wilke *et al.*, 2014).

Optimizing the efficacy of anti‐angiogenic therapies for the treatment of cancer has long been a subject of research. Already in 1972, the concept of vascular normalization was introduced in which they observed that tumor vessels obtained a more orderly arrangement following razoxane treatment (Le Serve and Hellmann, 1972). Decades later, it was proposed that careful scheduling of anti‐angiogenic therapy could restore the balance between pro‐ and anti‐angiogenic factors in tumor tissue, resulting in ‘normalized’ vasculature, that is, less leaky, dilated, and tortuous vessels (Jain, 2001). Supposedly, this normalized vasculature would result in increased tumor oxygenation and improved penetration of drugs. However, various studies that tested this hypothesis yielded contradictory results (Arjaans *et al.*, 2013; Chauhan *et al.*, 2012; Dickson *et al.*, 2007; Heskamp *et al.*, 2013; Tong *et al.*, 2004; Van der Veldt *et al.*, 2012; Vasudev and Reynolds, 2014). Therefore, it is now accepted that the effects of anti‐angiogenic therapy on tumor vasculature are highly dose‐, time‐, and tumor‐type dependent (Huang *et al.*, 2013; Smith *et al.*, 2013).

In light of this, we aimed to clarify the effects of anti‐angiogenic drug scheduling on hemodynamic function and penetration of drugs in EAC. In the present study, patient‐derived xenograft mouse models of EAC were subjected to long‐term and short‐term angiogenesis inhibition with a murine VEGFR2 inhibitor. Using a multi‐agent dynamic contrast‐enhanced (DCE‐) MRI method, we revealed that long‐term (LT) VEGFR2 targeting reduced intratumoral flow and vascular permeability resulting in reduced nanoparticle albumin‐bound (nab) paclitaxel (NPTX) uptake. Surprisingly, short‐term anti‐angiogenic therapy increased the vessel diameter and density and resulted in improved NPTX uptake. Furthermore, we identified the stromal mechanisms through which these effects are mediated and provide clues on how to improve the efficacy of prolonged anti‐angiogenic therapy combined with chemotherapy.

## 2 Materials and methods

### 2.1 Study approval

All patients included in the BiOES biobank signed informed consent according to the procedures approved by the Amsterdam UMC ethical committee (METC 01/288#08.17.1042). All pertinent procedures which are described in this manuscript, such as the collection and expansion of tissue in xenografts and cell lines, are covered by this informed consent. Grafting of EAC tumor pieces in mice, treatment with DC101 and nab‐paclitaxel, and MRI imaging were approved and performed according to local legislation and ethical approval and in accordance with ARRIVE guidelines (Amsterdam UMC protocols LEX100780, LEX102774, and LEX103159 and University Maastricht protocol 2014‐067).

### 2.2 Establishment of primary cultures

Primary cultures were established as described earlier (Damhofer *et al.*, 2015). Tumor material of patients diagnosed with EAC in the Amsterdam UMC, location AMC, was collected as described earlier and approved by the institute’s ethical committee and performed according to the guidelines of the Helsinki Convention (MEC 01/288#08.17.1042) (Damhofer *et al.*, 2015). Immunocompromised NOD.Cg‐Prkdc^scid^ Il2rg^tm1Wjl^/SzJ (NSG) mice were grafted subcutaneously with patient material for expansion. Mice were maintained and bred at the local animal facility in pathogen‐free cages with at least two to five cage companions, under the supervision and ethical approval of the Amsterdam animal experiment ethical committee (LEX100780, LEX102774, and LEX103159).

For isolation of either human tumor cells or murine cancer‐associated fibroblasts (CAFs) from harvested patient‐derived xenografts (PDX), cell sorting for EpCAM and H2Db was performed to obtain pure populations. The primary tumor cell culture used for *in vivo *studies is AMC‐EAC‐007B (007B), which was derived from a pretreatment biopsy. Murine endothelial cells (ECs), which were isolated from lungs, and murine CAFs were used for *in vitro* stimulations. Murine ECs were kindly provided by S. Tas (Amsterdam University Medical Center) using the following protocol. ECs were isolated by digesting minced lung tissue with collagenase, passing the solution through a 70‐µm cell strainer, and culturing the cells for 24 h in medium. Macrophages were removed from the culture using FCγRII/III antibody (BD553142, BD Biosciences, Franklin Lakes, NJ, USA, 1 : 300) and magnetic Dynabeads conjugated to sheep anti‐rat IgG (110‐35, Invitrogen, Carlsbad, CA, USA). Subsequently, ECs were isolated from the culture with Dynabeads and ICAM‐2 antibody (553326, BD Biosciences, 1 : 300). ECs were maintained in DMEM supplemented with 8% fetal bovine serum, L‐glutamine (2 mm), penicillin (100 units·mL^−1^), and streptomycin (500 μg·mL^−1^) (Lonza, Basel, Switzerland). 007B cells and fibroblasts were maintained in fully supplemented IMDM.

### 2.3 Reagents

DC101, a murine VEGFR2 inhibitor and a gift from ImClone Systems (Eli Lilly and Company, Indianapolis, IN, USA), and nab‐paclitaxel (NPTX, Abraxane, Celgene, Summit, NJ, USA) were reconstituted as described earlier (Steins *et al.*, 2017). For multi‐agent DCE‐MRI, the contrast agents were a high molecular weight (MW, 59 517 Da) generation 5 dendrimer (G5‐PPI‐(PEG_6_GdDOTA)_64_), an intermediate MW (7317 Da) generation 2 dendrimer (G2‐PPI‐(PEG_6_GdDOTA)_8_), and a low MW (754 Da) gadoterate meglumine (Gd‐DOTA, Dotarem®, Guerbet, Villepinte, France). High MW agents are sensitive to blood volume and vessel permeability changes, while low MW agents are sensitive to blood flow changes (Jaspers *et al.*, 2009; de Lussanet *et al.*, 2005). Dendrimer‐based contrast agents were manufactured by SyMO‐Chem BV (Eindhoven, The Netherlands) as described earlier (Hectors *et al.*, 2018).

For *in vitro* stimulation with DC101, cells were plated in starvation medium (0.5% FCS) and after overnight adhesion 50 nm DC101 was added to the culture medium for 3 days. For *in vitro* activation of murine fibroblasts, cells were placed in starvation medium (0.5% FCS) overnight when they had reached 70% confluency and subsequently 5 ng·mL^−1^ rTGF‐β (PeproTech, Rocky Hill, NJ, USA) was added to the medium for 24 h. For *in vitro* stimulation of murine ECs with rFGF2 (Tebu‐bio, Le Perray‐en‐Yvelines, France), cells were placed in starvation medium (0.1% FCS) overnight when they had reached 70% confluency and subsequently 10 ng·mL^−1^ rFGF2 was added to the medium for 24 h. After stimulation, cells were trypsinized for RNA isolation or fixed in 4% paraformaldehyde for immunofluorescent staining.

### 2.4 Animal study design and tumor inoculation

Female athymic nude Foxn1^nu^ mice were maintained and inoculated with 007B cells as described previously (Steins *et al.*, 2017). Tumor cells were injected on the right hind limb to prevent motion artifacts during MRI scanning. Animal welfare, weight, and tumor size were assessed twice a week. No adverse events were reported as a consequence of tumor growth, DC101, or nab‐paclitaxel treatment. Tumor‐bearing mice (~ 50–75 mm^3^) were randomly divided into short‐term (ST) and long‐term (LT) therapy groups (*n* = 10 per group). LT therapy groups were intraperitoneally injected with either DC101 (40 mg·kg^−1^) or PBS control (*n* = 5 per group) twice a week for 4 weeks. ST therapy groups received a single i.p. injection with either DC101 (40 mg·kg^−1^) or PBS control when the tumor was ~ 800 mm^3^, which was the average tumor size after LT therapy. For all groups, 3 days after the last injection of DC101 or PBS, mice were either injected with nab‐paclitaxel (NPTX) which was measured in the tumor using liquid chromatography/tandem mass spectrometry (see Supplementary Materials for a detailed description), or subjected to an MRI measurement followed by vascular casting and tumor harvesting.

### 2.5 Multi‐agent DCE and diffusion‐weighted MRI

Multi‐agent DCE and diffusion‐weighted MRI (DW‐MRI) were performed. Using differently sized contrast agents, vascular permeability could be quantified after treatment. DCE‐MRI was performed according to the protocol of Hectors and colleagues (Hectors *et al.*, 2018). Mice were anesthetized with isoflurane, and an infusion line containing the G5 dendrimer, G2 dendrimer, and Gd‐DOTA was placed in the tail vein. After being positioned in a custom‐made cradle, the tumor‐bearing right hind limb was fixated in alginate to minimize movement artifacts and MRI was performed. Respiration rate was monitored using a balloon pressure sensor, and temperature of the mouse was monitored with a rectal temperature probe and kept constant during the MRI acquisition by use of a heating pad in the cradle. MR imaging was performed on a 7T Bruker BioSpec 70/30 USR (Bruker BioSpin MRI GmbH, Ettlingen, Germany) using a 1H 59/35 mm (outer/inner diameter) circular polarized transceiver volume coil (Bruker BioSpin MRI GmbH). DCE‐MRI measurements and analyses (i.e., processing and fitting) were performed as previously described (Hectors *et al.*, 2018).

Prior to contrast injection, diffusion‐weighted images were acquired using a fat‐suppressed echo‐planar imaging (EPI) sequence with repetition time/echo time = 4000/18.5 ms, flip angle = 90, field of view = 60 × 30 × 24 mm^3^, acquisition matrix = 150 × 72 × 16, reconstructed to 150 × 75 × 16, and *b*‐values = 0, 2, 5, 10, 15, 20, 25, 50, 100, 200, 400, 600, and 800 s·mm^−2^ using 3 orthogonal gradient directions and 2 signal averages. A multi‐slice multi‐echo sequence with echo train length = 8–400 ms in steps of 8 ms, repetition time = 10 000 ms, flip angle = 90/180, field of view = 30 × 30 × 24 mm^3^, acquisition matrix = 39 × 50 × 16 reconstructed to 75 × 75 × 16 was used to calculate tissue T2 values by pixel‐wise fitting of S(TE) = S(0)exp(‐TE/T2) in MATLAB (R2016b, MathWorks, Natick, MA, USA).

Signal intensity at the acquired *b*‐values was fitted with the IVIM model using a nonlinear least‐squares fit in MATLAB. Tissues T1 and T2 were set voxel‐wise using the corresponding acquired T1 and T2 maps and the T1 and T2 of blood were set to 2200 ms and 50 ms, respectively (Lin *et al.*, 2012), to yield absolute parametric maps for tissue diffusivity (*D*), perfusion fraction (*f*), and the pseudodiffusion coefficient (D*) as described in formula 2 of Lemke *et al.* (2010). Median parameter values within the tumor were calculated for further analysis for the same voxels as used for the DCE‐MRI analysis.

Quantified contrast uptake curves were simultaneously fitted with the gamma capillary transit time model (Schabel, 2012). Fractional blood volume (*v*_b_), mean capillary transit time (*t*_c_), vascular heterogeneity index (α^−1^), interstitial space volume fraction (*v*_e_), and delay time between contrast agent injection and bolus arrival in the tumor (*t*_d_) were constrained to be identical between the boluses, while the extraction fraction (*E_n_*) was separately assessed for each agent. Overall, blood flow was calculated from model fit parameters (*F* = *v*_b_/*t*_c_), as were transfer constants for each contrast agent ($K_{n}^{trans}$ = *E_n_*·F). Median parameter values within the tumor were calculated after removing voxels with a goodness‐of‐fit *r* ^2^ < 0.9 from the analysis. By using the intravoxel incoherent motion (IVIM) model, the DW‐MRI was quantified to yield the tissue diffusivity (*D*), related to cell density, and perfusion fraction (*f*) without the use of contrast agents (Le Bihan *et al.*, 1988). As the extensive MRI protocol used in this study precluded repeated measures, MRI data could not be paired.

### 2.6 Vascular casting

Directly after DCE‐MRI, mice were kept under anesthesia and transferred to a heating pad. After intraperitoneal injection with 250 I.E. of heparin (LEO Pharma, Ballerup, Denmark), the abdomen was incised and left open using an Alm retractor (World Precision Instruments (WPI), Sarasota, Florida, USA). Intestines were moved aside to isolate the aorta and caval vein using curved forceps. Subsequently, two sutures were placed around the aorta and, using a Vannas scissors (WPI), a small incision was made in the abdominal aorta. The aorta was then cannulated with a handmade cannula, consisting of silicon infusion tubing (Instech, Plymouth Meeting, PA, USA) and a 26‐G needle of which the plastic end was burned off and the tip of the needle was made blunt with a file. Prior to cannulation, the silicon tubing was dipped in ether causing it to dilate allowing the blunted needle to be placed in the tubing that then shrunk again. On the other end of the silicon line, a 25‐G needle was inserted. The aorta was cannulated with the blunt‐end needle in the caudal direction, and the cannula was fixed in the aorta using the sutures. A syringe containing wash buffer (PBS + 1% heparin at 37 °C) was connected to the cannula. Subsequently, an incision was made in the vena cava and the vasculature was gently flushed with wash buffer for 30 min. The corrosion casting medium Mercox (Ladd Research, Williston, Vermont, USA) was labeled with UV‐blue fluorescent dye (vasQtec, Zürich, Switzerland, 0.275 mg·mL^−1^), prepared (3 mL resin + 25 µL catalyst) in a syringe, and the vasculature was slowly filled with Mercox at 100 mmHg using a handmade pressure system (Schwarz *et al.*, 2017). Forty minutes after infusion, Mercox had polymerized and the tumor‐containing right hind limb was harvested and fixed for 48 h in 4% PFA and equilibrated for 48 h in 20% sucrose in PBS at 4 ºC. The prepared hind limb was embedded in carboxymethylcellulose sodium solvent 5% (Brunschwig Chemie, Amsterdam, The Netherlands) and Indian ink 5% (Royal Talens, Apeldoorn, The Netherlands), and stored at −20 ºC for further processing.

The frozen specimens were processed by alternately cutting 28‐µm slices followed by episcopic imaging. After each cut, a fluorescence image in the Mercox channel was acquired with peak excitation at 365 nm and emission at 505 nm, an exposure time of 20 000 ms, and a bin size of 1 × 1. Coregistered reflection images were obtained at 577–20 nm (ex) and 577 nm (em) with an exposure time of 200 ms and a bin size of 2 × 2. Digital images were 4096 × 4096 pixels at a bin size of 1 × 1, and the nonisotropic voxels (14 × 14 × 28 µm) were transferred into isotropic voxels measuring 28 × 28 × 28 µm. The coregistered fluorescence image stack yielded a 3D reconstruction of the vasculature, while reflection images resulted in an outline representation of the tumor. Images were further processed using Fiji (ImageJ v1.51n) (Schindelin *et al.*, 2012, 2015). Vessel density was calculated as the number of vessel segments per mm^3^. Vascular volume fraction was calculated as the total amount of volume detected as vessel divided by the total tumor volume. To quantify the Mercox images, 2D ROIs were drawn around the tumor for several slices with a regular interval and interpolated using the ROI Manager to restrict the 3D images to only comprise tumor. This volume was fed into the TubeAnalysis (Tischer and Tosi, 2016) batch process described with the following parameters: CloseRadiusScale = 14 µm, VesselRadius = 14 µm, VesselThreshold = 6, VesselVolumeThreshold = 500, and PruneEnds = false. The resulting vessel map was used to calculate the vascular volume fraction as the total amount of volume detected as vessel divided by the total tumor volume. Next, the vessel map was skeletonized using the Skeletonize plugin and the 3D skeleton image of the vessel bed was then analyzed using the AnalyzeSkeleton (2D/3D) (Arganda‐Carreras *et al.*, 2010) plugin to retrieve the number of separate vessel segments, calculating the vessel density as the number of vessel segments per mm^3^. To check whether the Mercox filling successfully penetrated the entire tumor, the Mercox‐derived vascular volume fraction was compared to the perfused percentage of the DCE‐MRI analysis (*r* ^2^ ≥ 0.9). In three tumors, the Mercox‐perfused fraction was markedly lower than for the MRI, and these tumors were excluded from further analysis.

#### 2.6.1 Nab‐paclitaxel injection and liquid chromatography/tandem mass spectrometry

Three days after the last injection of DC101 or PBS, NPTX (120 mg·kg^−1^) or saline control was administered via the tail vein. Two hours after NPTX administration, mice were anesthetized using isoflurane and sacrificed by heart puncture. Blood was collected in BD Vacutainer® K2 EDTA blood collection tubes which were spun down at 1300 RCF for 10 min, and plasma was stored at −80 °C. The tumor was harvested, and pieces of the tumor were either fixed in 4% paraformaldehyde (PFA) or snap‐frozen and stored at −80 °C until further processing. For analysis of intratumoral NPTX uptake following ST DC101, 3 of 5 mice had to be excluded due to failed intravenous NPTX injection in which both plasma and tumor concentrations were too low to analyze. Snap‐frozen tumor samples were processed and measured with liquid chromatography/tandem mass spectrometry (LC‐MS/MS) as described earlier (Steins *et al.*, 2017). To correct for injection error, tumor NPTX concentrations were normalized against the plasma NPTX concentration of each mouse (i.e., relative NPTX uptake).

#### 2.6.2 Immunohistochemistry

After fixation, dehydration, and embedding of the tumor tissue, 4‐µm‐thick slices were cut using a microtome. Tissue sections from control and DC101‐treated PDX tumors were deparaffinized and rehydrated in a series of ethanol. Alcian blue and picrosirius red (PSR) staining were performed according to standard protocols. For immunohistochemistry (IHC), endogenous peroxidase was blocked using 0.3% hydrogen peroxide (VWR International, Radnor, PA, USA) in methanol for 15 min at RT. Subsequently, heat‐induced epitope retrieval (HIER) and overnight incubation of the primary antibody diluted in antibody diluent (Klinipath, Radnor, PA, USA) at 4 °C were performed as indicated in Table S1. The following day, BrightVision + post‐antibody block was applied on the sections for 20 min at RT followed by secondary antibody BrightVision Poly‐HRP‐anti Ms/Rb IgG (both Immunologic, Radnor, PA, USA, VWR International) for 30 min at RT. For HA staining, a rabbit anti‐sheep antibody (6016‐01, Southern Biotech, Birmingham, AL, USA, 1 : 3000) step was performed for 30 min at RT prior to BrightVision Poly‐HRP‐anti Ms/Rb IgG. Staining was developed using Bright‐DAB (Immunologic) and counterstained with hematoxylin (Klinipath). Sections were dehydrated in a series of alcohol and xylene and mounted in Pertex (Histolab, Askim, Sweden). For quantification of staining using imagej software (San Diego, CA, USA), hematoxylin and DAB staining was separated using the color deconvolution plugin in the H‐DAB channel. For PSR separation, the RGB channel was used, and for Alcian blue separation, the H&E channel was used. The same threshold was set for each image, and the region of interest was selected using the ROI manager tool. For quantifying the percentage of Ki67‐positive cells, the amount of DAB‐positive nuclei was divided by the amount of nuclei as measured with hematoxylin. For vessel lumen quantification, separate ROIs were drawn within each vessel and the ROI surface was calculated as percentage of area.

#### 2.6.3 Quantitative RT‐PCR

RNA was isolated (Macherey‐Nagel, Bioké, Düren, Germany), cDNA was synthesized using SuperScript III (Invitrogen), and quantitative RT‐PCR was performed with SYBR Green (Roche, Basel, Switzerland) on a LightCycler 480II (Roche), all according to manufacturer’s instructions. The delta threshold cycle (*C*_t_) was determined by normalizing values to B2M. The primers that were used in this study are listed in Table S2.

#### 2.6.4 Immunofluorescence

mCherry‐labeled murine fibroblasts were plated in monoculture or in coculture with Cerulean‐labeled murine ECs on glass coverslips in starvation medium (IMDM + 0.5% FCS). The following day, 50 nm DC101 or PBS control was added to the cultures. Three days later, cells were fixed in 4% paraformaldehyde for 10 min, permeabilized with 1% Triton X‐100 (Sigma, St. Louis, MI, USA) in PBS for 10 min, and incubated in blocking buffer (5% normal goat serum (Abcam, Cambridge, UK) in 0.1% Triton X‐100/PBS) for 30 min. Primary antibody directed against α‐SMA (ab5694, Abcam, 1 : 100 in blocking buffer) was incubated for 2 h at room temperature (RT). Secondary antibody labeled with Alexa Fluor 488 (A11008, Thermo Fisher Scientific, Waltham, MA, USA, 1 : 300 in blocking buffer) was incubated for 1 h at RT in the dark. Nuclei were stained with Hoechst (Thermo Fisher Scientific, 1 : 1000 in PBS) for 5 min. Coverslips were mounted on glass slides with ProLong Gold Antifade Reagent (Life Technologies, Carlsbad, CA, USA) and imaged on a confocal SP8‐X DLS microscope (Leica, Wetzlar, Germany).

#### 2.6.5 Gene correlations

To identify the most significantly correlated genes to a single gene, the R2: Genomics Analysis and Visualization Platform was used (http://r2.amc.nl). The Cancer Genome Atlas (TCGA) study containing esophageal carcinoma tissue (study ID: TCGA‐ESCA; https://portal.gdc.cancer.gov/projects/TCGA-ESCA) was selected to correlate *HYAL2 *expression to stromal activation genes and the most significant correlated genes to *NOS3*. The TCGA study and Barbour study (study ID: http://www.ncbi.nlm.nih.gov/geo/query/acc.cgi?acc=gse72872; https://www.ncbi.nlm.nih.gov/geo/query/acc.cgi?acc=gse72872) were used to identify genes correlated with *NOS3* expression using gene category ‘drug target’ and KEGG pathway ‘pathways in cancer’. All analyses were performed on EAC samples only.

### 2.7 Statistical analysis

Statistical significance between groups was determined using Mann–Whitney *U*‐test of Student’s *t*‐test. Statistical analysis on growth curves was assessed using two‐way repeated measures ANOVA. *Z*‐scores of MRI and Mercox parameters were determined by normalization of each experimental mouse to the average and standard deviation of control mice. Pearson’s correlation coefficients were calculated to determine *P*‐values in gene expression data. Bar graphs and dot plots with error bars represent mean and ±SD, respectively. Box plots represent minimum, first quartile, median, third quartile, and maximum. Statistical analyses were performed using graphpad prism 6 (San Diego, CA, USA) and ibm spss statistics for Windows, version 25.0 (IBM Corp, Armonk, NY, USA). Tests were performed two‐tailed, and a *P*‐value < 0.05 was considered statistically significant.

## 3 Results

### 3.1 Short‐term anti‐angiogenic therapy dilates the tumor vasculature and improves intratumoral drug delivery, while prolonged angiogenesis inhibition reduces vasculature and impedes drug delivery

**Figure 1:**
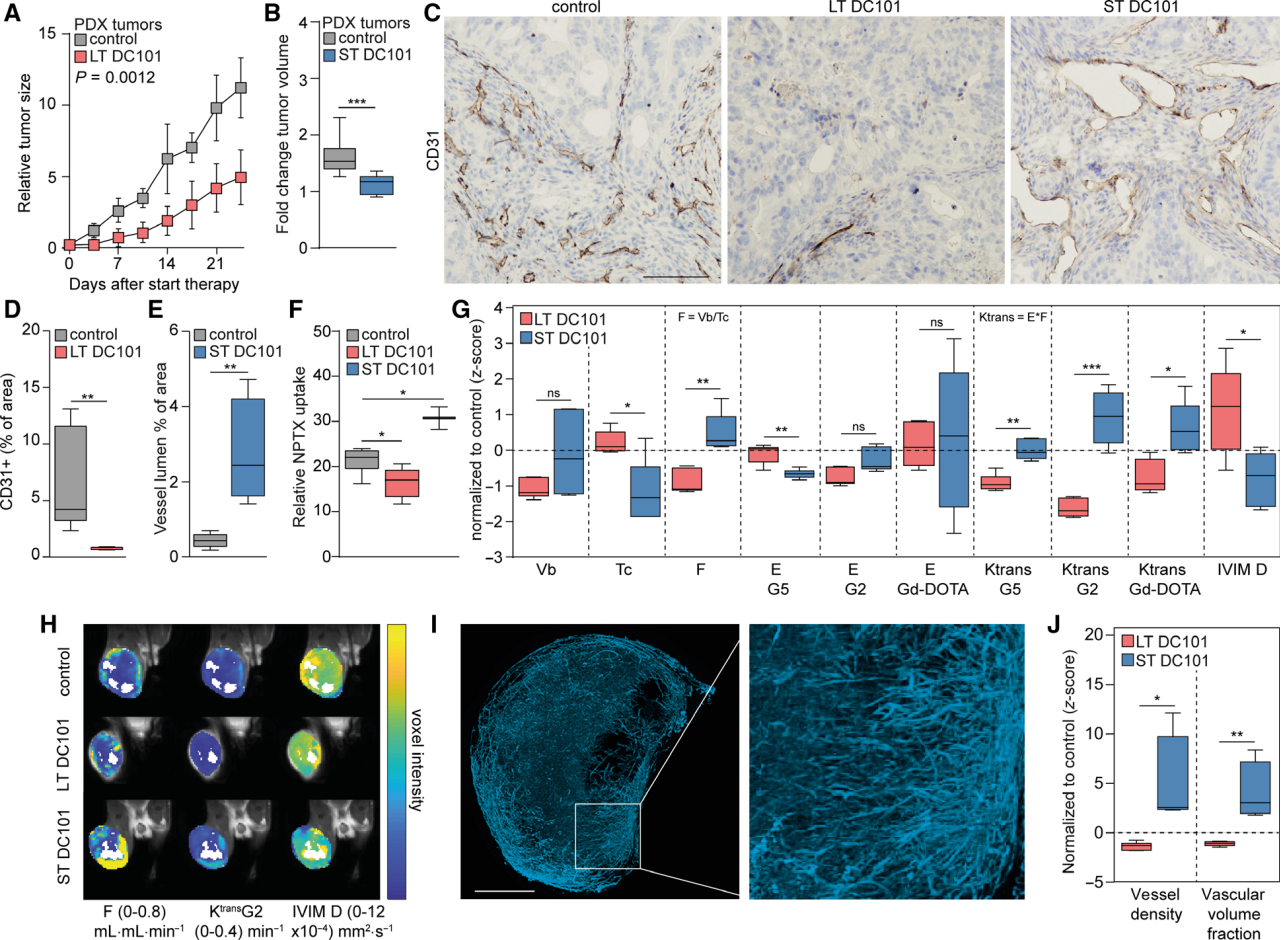
Short‐term anti‐angiogenic therapy dilates the tumor vasculature and improves intratumoral drug delivery. (A) Nude mice with subcutaneous EAC‐derived PDX tumors were injected with DC101 (40 mg·kg^−1^) or PBS twice a week for 4 weeks. Tumor growth was normalized against the tumor size at the start of the experiment. *n* = 5 per group. Two‐way repeated measures ANOVA. (B) Nude mice with subcutaneous EAC‐derived PDX tumors that reached 800 mm^3^ were injected with DC101 (40 mg·kg^−1^) or PBS. Three days later, the tumors were harvested and tumor volume was normalized against the tumor size at the injection day, *n* = 11 per group. Student’s *t*‐test. (C) Tumor vascularization was stained with anti‐CD31 antibody using IHC. Scale bar is 100 µm. (D) Quantification of CD31‐positive cells as percentage of area in tumor sections using ImageJ software, *n* = 5 per group. Student’s *t*‐test. (E) Quantification of vessel lumen as percentage of area in tumor sections using ImageJ software, *n* = 5 per group. Student’s *t*‐test. (F) Intratumoral NPTX concentration normalized against plasma NTPX concentration. *n* = 8 for control group, and *n* = 5 for LT and *n* = 2 for ST DC101 groups. Mann–Whitney test. (G) Multi‐agent DCE‐MRI parameters in LT and ST DC101‐treated tumors, normalized to control. *n* = 15 for LT and *n* = 5 for ST control groups, and *n* = 5 for DC101 groups. Student’s *t*‐test or Mann–Whitney test. (H) Parameter maps overlaid on the MRI images. (I) Maximum intensity projection of the filled vasculature of a control tumor with fluorescently labeled Mercox. Scale bar is 250 µm. (J) Quantification of the Mercox fluorescent images assessing tumor vessel density and vascular volume fraction, normalized to control. *n* = 5 for LT and *n* = 4 for ST control groups, and *n* = 6 for LT and *n* = 4 for ST DC101 groups. Student’s *t*‐test. Error bars in all bar graphs represent SD. *P < 0.05, **P < 0.01, ***P < 0.001.

To study the effects of anti‐angiogenic drug scheduling on hemodynamic function and penetration of drugs in EAC, tumor‐bearing immunodeficient mice received long‐term treatment (LT) or short‐term treatment (ST) (Fig. S1A). Subsequently, mice were subjected to either intravenous nab‐paclitaxel injection (Fig. S1B) or multi‐agent DCE‐MRI and fluorescent casting of the tumor vasculature (Fig. S1C–E). LT anti‐VEGFR2‐treated tumors showed a 50% growth reduction compared to control (Fig. 1A), which was accompanied by a reduction in the number of CD31‐positive cells (Fig. 1C,D). Further analysis of Ki67 and cleaved caspase‐3 showed that apoptosis increased, explaining how anti‐VEGFR2 impedes tumor growth (Fig. S2A–D). Subsequent assessment of intratumoral NPTX by LC‐MS/MS demonstrated a significant reduction in chemotherapy uptake after LT VEGFR2 targeting compared to control (Fig. 1F). Assessment of ST anti‐VEGFR2‐treated tumors revealed that tumor growth was fully arrested after 3 days (Fig. 1B), which not be explained by changes in proliferation or apoptosis (Fig. S2A–D). Notably, IHC staining of the endothelium using anti‐CD31 showed a strongly dilated vasculature (Fig. 1C,E) and an increased intratumoral delivery of NPTX as determined by LC‐MS/MS (Fig. 1F).

To identify mechanisms that could explain the reduced intratumoral delivery of chemotherapy after LT treatment and—more importantly—increased chemotherapy delivery after ST treatment, hemodynamics of the tumors was assessed by multi‐agent DCE‐MRI. This revealed opposite effects in the majority of hemodynamic MRI parameters following LT or ST VEGFR2 inhibition (Fig. 1G). The intratumoral blood flow (*F*), which is defined by *v*_b_/*t*_c_, was relatively lower following LT VEGFR2 inhibition, while this was relatively higher after ST VEGFR2 inhibition (Fig. 1G,H). Since no significant changes between LT and ST treatment were found in the blood volume fraction (*v*_b_), the relatively higher F following ST treatment was explained by a relatively lower capillary transit time (*t*_c_). Moreover, the extraction fraction (*E*), of the G5 contrast agent, which reflects vascular permeability, was relatively lower by ST VEGFR2 inhibition. Extraction fractions for the G2 and Dotarem contrast agents were not affected by anti‐angiogenic treatment, suggesting that vessels become less permeable for high molecular weight agents only. The volume transfer constant (*K* ^trans^), which is the composite measure of *E* and *F*, of all contrasting agents was relatively lower after LT VEGFR2 inhibition and relatively higher after ST inhibition, of which the largest changes were observed in the G2 contrast agent (Fig. 1G,H). Since the extraction fraction was largely unaffected by ST or LT VEGFR2 inhibition, these changes in *K* ^trans^ are mainly explained by blood flow changes following anti‐angiogenic treatment. Opposite effects were also observed in the diffusion (IVIM D), in which a higher diffusion is associated with a lower intratumoral cellular density. This suggests a relatively lower intratumoral cellular density (i.e., number of cells per area) after LT VEGFR2 inhibition and a relatively higher cellular density following ST anti‐angiogenic treatment (Fig. 1G,H). Subsequent vascular casting of the tumors with Mercox fluorescent resin (Fig. 1I) supported these observations; LT VEGFR2 inhibition resulted in a relatively lower vessel density and volume, while these parameters were relatively higher following ST VEGFR2 inhibition (Fig. 1J).

Together, these results show that LT VEGFR2 inhibition leads to significant vascular regression, relatively lower flow and cellular density, and increased apoptosis resulting in delayed tumor growth but also reduced intratumoral delivery of chemotherapy. On the contrary, ST VEGFR2 inhibition rapidly increases the tumor vessel lumen and intratumoral flow resulting in improved intratumoral chemotherapy delivery in EAC, while tumor growth is arrested.

### 3.2 Short‐term anti‐angiogenic therapy activates cancer‐associated fibroblasts and drives NO synthesis

**Figure 2:**
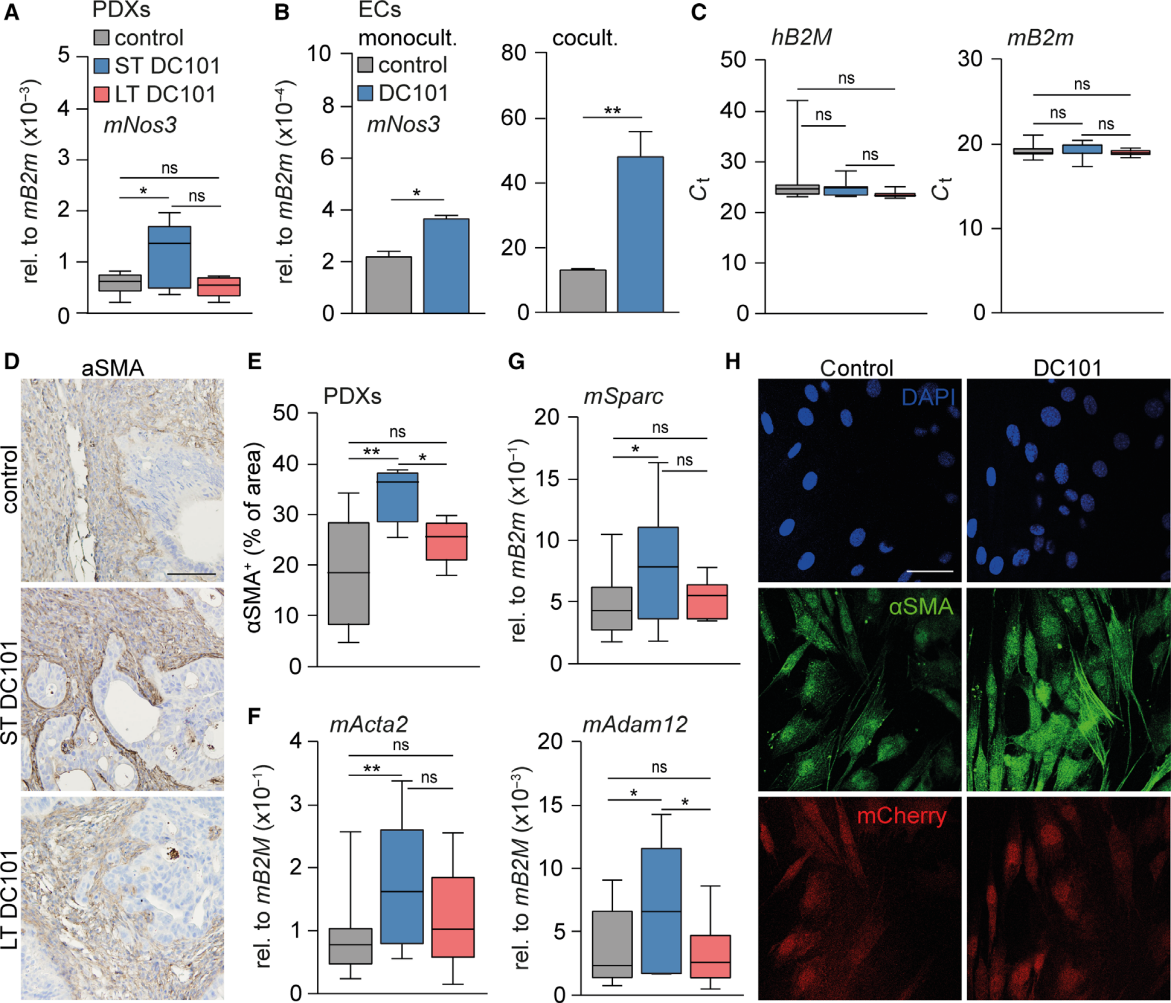
Short‐term anti‐angiogenic therapy activates cancer‐associated fibroblasts. (A) mRNA expression of stromal *mNos3* was determined in PDX tumors using qPCR. *n* = 10 for control group and *n* = 5 for LT and ST DC101 groups. (B) mRNA expression of *mNos3* was determined in ECs that were monocultured or cocultured with CAFs for 3 days using qPCR. *n* = 2. (C) As for panel A, assessing tumor beta‐2 microglobulin (*hB2M*) and stroma beta‐2 microglobulin (*mB2m*). *n* = 20 for control group and *n* = 10 for LT and ST DC101 groups. (D) PDX tumors were stained for alpha‐smooth muscle actin (α‐SMA) with IHC. Scale bar is 100 µm. (E) Quantification of α‐SMA as percentage of area using imagej software. *n* = 10 for control group and *n* = 5 for LT and ST DC101 groups. (F, G) As for panel A, assessing stromal *mActa2*, *mSparc,* and *mAdam12*. (H) mCherry‐labeled CAFs were cocultured with Cerulean‐labeled ECs, treated with DC101 or control for 3 days, and stained for α‐SMA with immunofluorescence. Scale bar is 50 µm. Error bars in all bar graphs represent SD. All Student’s *t*‐test. *P < 0.05, **P < 0.01.

Subsequently, we set out to elucidate the mechanisms that underlie the dilated vasculature and, ultimately, the enhanced chemotherapy uptake following ST VEGFR2 inhibition. Nitric oxide (NO) is an important factor in vasodilation (Durán *et al.*, 2013). NO regulates the vascular tone, and synthesis of NO is mostly mediated via endothelial nitric oxide synthase (eNOS or *NOS3*; Luiking *et al.*, 2010). Moreover, eNOS and inducible nitric oxide synthase (iNOS) play an important role in tumor angiogenesis, vascular tone, and growth (Clemons *et al.*, 2010; Cullis *et al.*, 2006; Fukumura and Jain, 1998; McAdam *et al.*, 2012; Vannini *et al.*, 2015; Ying and Hofseth, 2007). As the vascular effects mediated via iNOS are driven by inflammatory mediators and immunodeficient mice were used, we hypothesized that the observed vascular changes following ST VEGFR2 targeting might be mediated via eNOS. *In silico* assessment of a publicly available gene expression set containing non‐pretreated resected EAC samples (Cancer Genome Atlas Research Network *et al.*, 2017) showed that genes involved in angiogenesis (i.e., *ENG*, *CERCAM,* and *CD248*) correlated most strongly to *NOS3,* underscoring that expression of *NOS3* is restricted to the endothelium (Fig. S3A). Analysis of *mNos3* in our PDX tumors revealed that transcription of this synthase was upregulated after ST anti‐VEGFR2 therapy compared to control, and returned to baseline levels after LT treatment, suggesting that VEGFR2 inhibition indeed regulates NO production (Fig. 2A).

As DC101, a murine VEGFR2 inhibitor, cannot target the human tumor cells in PDXs, but can target both murine ECs and CAFs, we explored whether DC101 acts directly on ECs and *mNos3* transcription or whether CAFs play a role in this. Immunohistochemical analysis revealed that VEGFR2 expression was not restricted to the endothelium but also abundantly present in the tumor stroma (Fig. S3B). Cerulean‐labeled murine ECs were subjected to VEGFR2 inhibition for 3 days either in monoculture or in coculture with mCherry‐labeled murine CAFs (Fig. S3C). qRT‐PCR analysis showed that VEGFR2 inhibition increased *mNos3* expression in monocultured ECs. This effect was strongly enhanced when ECs were cocultured with CAFs (Fig. 2B). To determine whether the observed enhanced NO signaling was driven by an increase in CAFs, we quantified the murine host cells that make up the stroma in the PDXs. This showed similar amounts of human β2 microglobulin (*hB2M*) and mouse β2 microglobulin (*mB2m*) in all tumors (Fig. 2C), suggesting that the amount of stroma was not affected by inhibition of VEGFR2.

In contrast, the activation status of the stroma in the PDXs was strongly increased following ST VEGFR2 inhibition as determined by alpha‐smooth muscle actin (α‐SMA) protein expression (Fig. 2D,E). This was supported by transcript analysis of markers for stromal activation, such as alpha‐smooth muscle actin (*mActa2*)*,* secreted protein acidic and rich in cysteine (*mSparc*), and disintegrin and metalloproteinase domain‐containing protein 12 (*mAdam12*; Veenstra *et al.*, 2018; Fig. 2F,G). Remarkably, markers for stromal activation were similar to control after LT VEGFR2 targeting. Subsequently, mCherry‐labeled murine CAFs were subjected to VEGFR2 inhibition for 3 days either in monoculture or in coculture with Cerulean‐labeled murine ECs and fluorescently stained for α‐SMA. This revealed that VEGFR2 inhibition was not able to activate CAFs in monoculture (Fig. S3D) but only when cocultured with ECs (Fig. 2H), suggesting that inhibition of VEGFR2 activates the stroma via the endothelium. Given that the endothelium is significantly reduced in tumors after LT VEGFR2 targeting, this explains why stromal activation cannot be maintained. Together, these results show that a single dose of anti‐angiogenic therapy is able to affect the entire tumor microenvironment in a timeframe of just 3 days.

**Figure 3:**
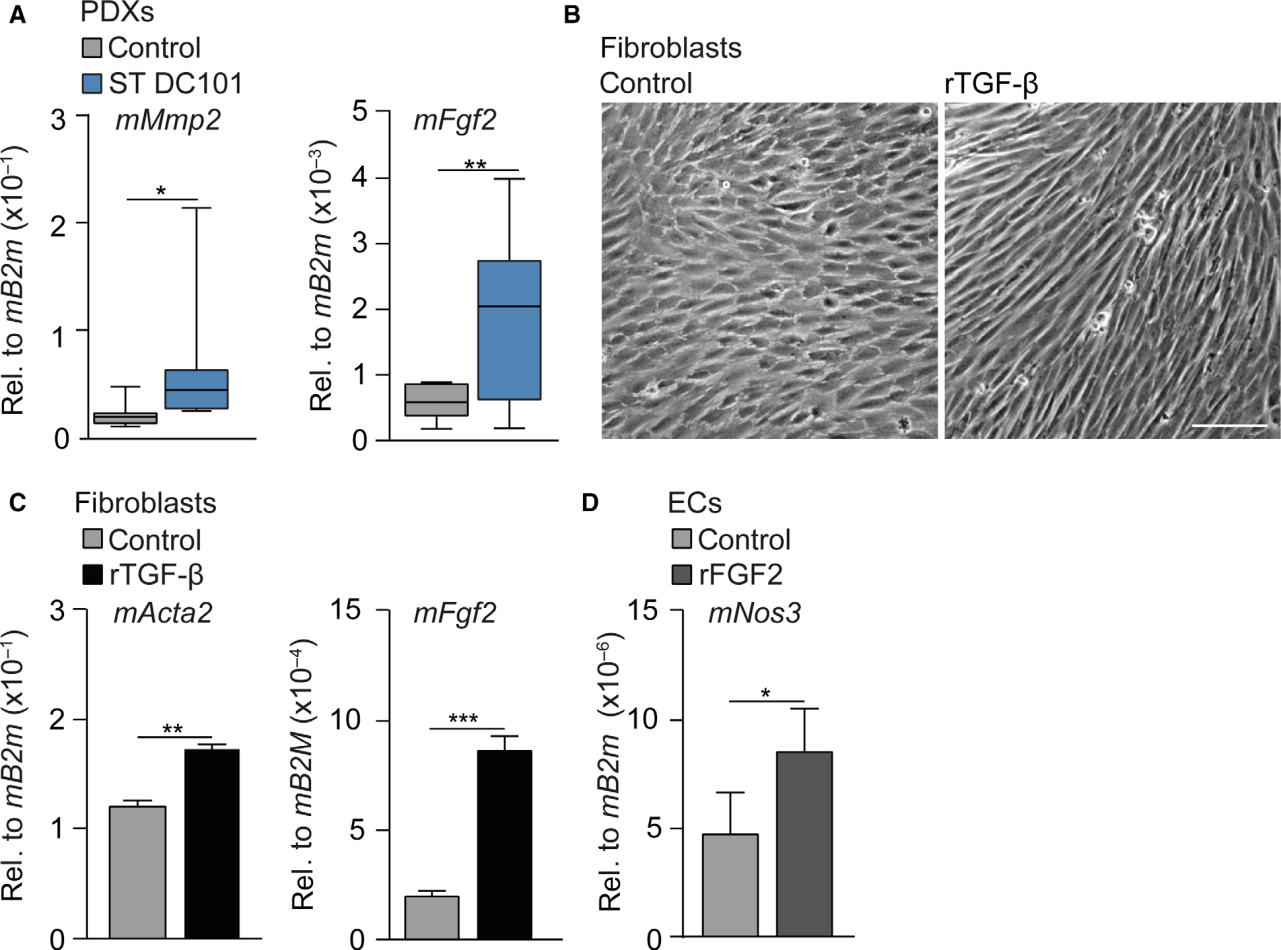
Activated stroma dilates the vasculature by driving NO synthesis. (A) mRNA expression of stromal *mMmp2* and *mFgf2* was determined in PDX tumors using qPCR. *n* = 10 per group. (B) Bright‐field images of murine CAFs treated with recombinant TGF‐β for 24h or left untreated. Scale bar is 100 µm. (C) As for panel A, assessing stromal *mActa2* and *mNos3*. *n* = 3 per group. (D) mRNA expression of *mNos3* was determined in ECs that were treated with rFGF2 for 24h or left untreated using qPCR. *n* = 3. *P < 0.05, **P < 0.01, ***P < 0.001. Error bars in all bar graphs represent SD. All Student’s *t*‐test.

As ST VEGFR2 inhibition activated CAFs, we reasoned that these CAFs produce a factor that stimulates *mNos3* transcription in ECs. To identify this factor, we queried genes that positively correlate to *NOS3* expression in the TCGA (Cancer Genome Atlas Research Network *et al.*, 2017) and another publicly available dataset (Krause *et al.*, 2016), both containing non‐pretreated resected EAC samples. Three genes were identified in both sets of which matrix metalloproteinase‐2 (*MMP2*) and fibroblast growth factor 2 (*FGF2*) were the most likely candidates as they are both secreted by CAFs (Tables S3 and S4). Of these candidates, expression of *mFgf2* was increased most after ST VEGFR2 inhibition (Fig. 3A) and we tested whether secretion of *mFgf2* was increased after stromal activation. Murine CAFs that were activated by adding recombinant TGF‐β (rTGF‐β) to the cultures obtained an elongated morphology (Fig. 3B) and expressed the stromal activation marker *mActa2.* Indeed, *mFgf2* was also upregulated (Fig. 3C). Subsequently, recombinant FGF2 was added to EC cultures for 24 h and mRNA levels of *mNos3* were found to be significantly upregulated in response to this ligand (Fig. 3D). Thus, stromal activation after short‐term VEGFR2 inhibition leads to increased expression of proteins like FGF2 by CAFs, and such ligands drive NOS3 expression and NO signaling resulting in vasodilation and enhanced chemotherapy delivery.

### 3.3 Activated CAFs degrade the ECM by expression of hyaluronidase‐2

**Figure 4:**
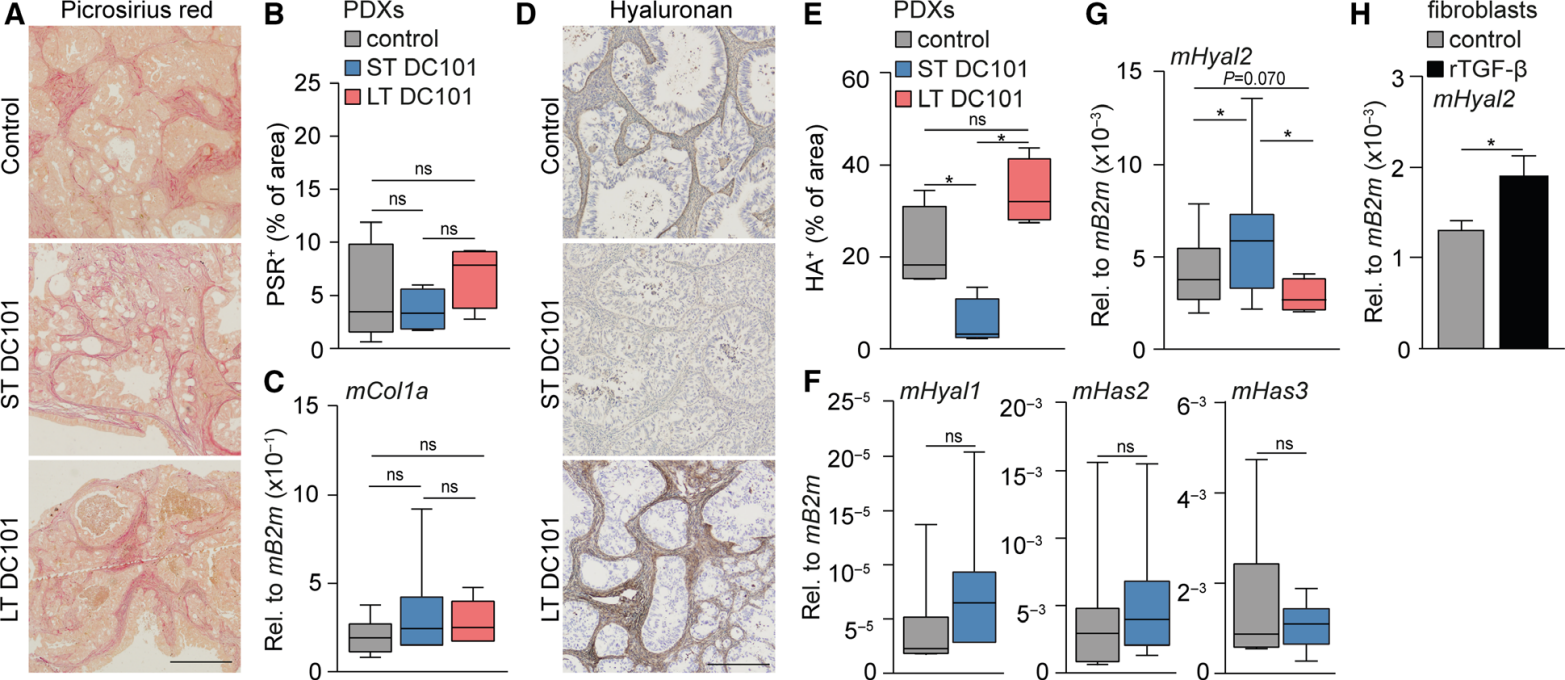
Activated CAFs degrade the ECM via expression of hyaluronidase‐2. (A) PDX tumors were histochemically stained for collagens with picrosirius red (PSR). Scale bar is 500 µm. (B) Quantification of PSR as percentage of area using ImageJ software. *n* = 9 for control group and *n* = 4 for LT and ST DC101 groups. (C) mRNA expression of stromal *mCol1a* was determined in PDX tumors using qPCR. *n* = 20 for control group and *n* = 10 for LT and ST DC101 groups. (D) PDX tumors were stained for hyaluronan (HA) with IHC. Scale bar is 250 µm. (E) Quantification of HA as percentage of area using imagej software. *n* = 5 for control group and *n* = 4 for LT and ST DC101 groups. (F) As for panel C, assessing stromal *mHyal1*, *mHas2,* and *mHas3*. *n* = 10 per group. (G) As for panel C, assessing stromal *mHyal2*. *n* = 20 for control group and *n* = 10 for LT and ST DC101 groups. (H) mRNA expression of *mHyal2* was determined in CAFs that were treated with rTGF‐β for 24 h or left untreated using qPCR. *n* = 3. Error bars in all bar graphs represent SD. All Student’s *t*‐test. *P < 0.05.

While the enhanced NO synthesis can explain the vasodilation after ST VEGFR2 inhibition, the mechanism behind the observed increase in intratumoral cellular density and arrested tumor growth remained to be elucidated. As markers for proliferation and apoptosis were unchanged after ST VEGFR2 targeting, this cannot explain changes in tumor cellularity. The observed increases in *K* ^trans^, however, could be mediated by changes in interstitial fluid pressure (IFP) which could hint at a role for the extracellular matrix (ECM; Hompland *et al*. 2013) to explain increased intratumoral cellular density and arrested tumor growth. For instance, changes in collagen content are known to affect tumor volume and cellular density (Stylianopoulos *et al.*, 2012; Voutouri *et al.*, 2016). However, microscopic (Fig. 4A,B) and transcript analysis (Fig. 4C) of collagens (picrosirius red staining) revealed no differences in control‐, ST‐, and LT‐treated tumors, suggesting that changes in collagens are not the cause of the observed effects of ST VEGFR2 inhibition (Fig. 4C).

Another ECM component that defines the mechanical properties of a tumor is hyaluronan (HA), which is known to increase IFP and thereby affect tumor volume and cellular density (Stylianopoulos *et al.*, 2012; Voutouri *et al.*, 2016). Evaluation of the stroma‐rich control tumors showed a high amount of HA (Fig. 4D). Subsequent analysis of LT and ST anti‐VEGFR2‐treated tumors revealed that HA was also abundantly present in LT‐treated tumors, while HA was completely absent after ST therapy (Fig. 4D,E). This was supported by IHC staining of Aggrecan, a water‐binding proteoglycan that forms a complex with HA (Fig. S4A,B). Alcian blue staining further confirmed the absence of proteoglycans after ST VEGFR2 inhibition (Fig. S4C,D). To determine whether these changes are caused by a reduced synthesis, or rather an increased degradation of HA, we assessed HA synthases (*mHas2* and *mHas3*) and hyaluronidases (*mHyal1* and *mHyal2*) in the stroma by qRT‐PCR. ST VEGFR2 inhibition did not affect HA synthases (Fig. 4F), but *Hyal2* was significantly upregulated following ST treatment resulting in a net degradation of HA (Fig. 4G). In contrast, *Hyal2* expression was significantly downregulated after LT VEGFR2 inhibition compared to ST inhibition and even substantially lower than control tumors (*P* = 0.070) (Fig. 4G).

**Figure 5:**
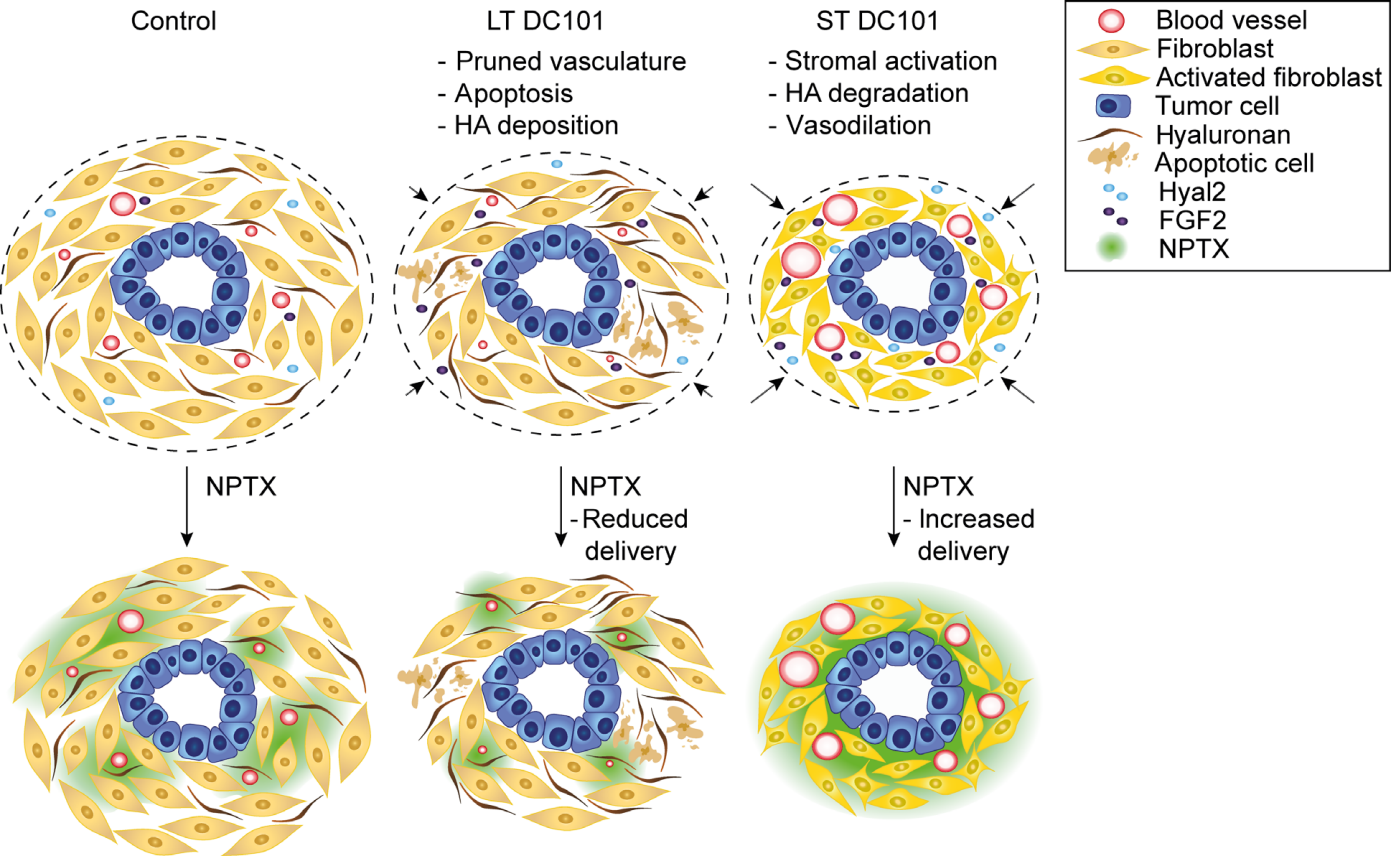
ST DC101‐induced NO synthesis and HA degradation improve chemotherapy delivery in EAC. LT DC101 treatment results in delayed tumor growth, as well as reduced NPTX uptake caused by a pruned vasculature and increased apoptosis and HA deposition. ST DC101 activates the CAFs which enhance the secretion of HYAL2 and FGF2 thereby degrading HA and increasing NO production resulting in vasodilation and improved chemotherapy delivery.

As ST VEGFR2 inhibition leads to stromal activation, we hypothesized that activated CAFs might drive *Hyal2* expression. *In silico* assessment of genes correlated with *HYAL2* in the TCGA dataset revealed strong positive correlations with stromal activation markers such as *ACTA2, SPARC, ADAM12,* and *FN1* (Fig. S4E). In order to show that activated CAFs can be a source of hyaluronidase‐2, we activated murine CAFs with rTGF‐β which indeed upregulated the transcription of hyaluronidase‐2 (Fig. 4H). Thus, we propose that the expression of *Hyal2* by activated CAFs is responsible for the degradation of HA. Since HA binds water molecules, degradation of HA—and thereby resolution of tumor edema—is likely to explain the rapid reduction in tumor volume and the increased intratumoral cellular density following ST VEGFR2 inhibition, while contributing to vascular decompression (Jacobetz *et al.*, 2013; Provenzano *et al.*, 2012). Thus, both enhanced NO synthesis and HA degradation following ST anti‐angiogenic treatment result in improved perfusion and chemotherapy delivery, and therefore provide new therapeutic leads to improve the efficacy of anti‐angiogenic therapies combined with chemotherapy (Fig. 5).

## 4 Discussion

Ramucirumab and paclitaxel are used for the treatment of advanced EAC, but survival benefits are marginal. Here, we investigated methods to improve the efficacy of this combination therapy and showed that LT VEGFR2 inhibition impaired the vasculature in PDXs of EAC resulting in decreased intratumoral NPTX delivery. On the contrary, ST anti‐VEGFR2‐treated tumors rapidly obtained more dilated vasculature resulting in improved chemotherapy delivery. We found these favorable effects to be mediated by stromal activation in which enhanced stromal secretion of HYAL2 and FGF2 led to HA degradation and NO signaling, respectively.

The effectiveness of the prolonged use of anti‐angiogenic therapies has been questioned for some time. Several preclinical studies have shown that prolonged angiogenesis inhibition results in an imbalance in pro‐ and anti‐angiogenic factors leading to pruned vasculature and decreased uptake of cytotoxic agents (Cesca *et al.*, 2016; Heskamp *et al.*, 2013; Jain, 2001). In addition, resistance to anti‐angiogenic therapy emerges, eventually even facilitating tumor invasiveness and metastatic growth (Ebos *et al.*, 2009). Our results are in line with these findings, showing that LT VEGFR2 inhibition pruned the vasculature and hampered the delivery of chemotherapy, and could possibly explain why survival benefits of ramucirumab with paclitaxel in EAC patients are limited. In contrast, in ST anti‐VEGFR2‐treated tumors chemotherapy uptake is enhanced, and similar findings have been reported in several other preclinical studies (Chauhan *et al.*, 2012; Dickson *et al.*, 2007; Tong *et al.*, 2004; Yang *et al.*, 2018). However, a single bolus of a VEGFR2 inhibitor in combination with a cytotoxic agent will be insufficient to improve treatment outcome in the clinical setting. Also, even when carefully sequencing anti‐angiogenic treatment with cytotoxic treatment to find a balance between pro‐ and anti‐angiogenic factors, ultimately ‘long‐term’ use of anti‐angiogenic agents will be needed in clinical practice to impact treatment outcome. Therefore, we set out to elucidate the mechanisms of increased chemotherapy uptake after a single bolus of a VEGFR2 inhibitor to find new therapeutic clues that could potentially improve the outcome of long‐term anti‐angiogenic treatment.

Previous studies have mainly focused on the effects of anti‐angiogenic therapy on ECs. Recently, it has become more evident that the ECM, and specifically CAFs, plays a key role in the induction of resistance to anti‐angiogenic therapy by production of a variety of growth factors that stimulate angiogenesis (Bhowmick *et al.*, 2004; Erez *et al.*, 2010). Several studies have identified escape mechanisms provided by the tumor stroma to circumvent angiogenesis inhibition via CAFs (Crawford *et al.*, 2009; Francia *et al.*, 2009; Rahbari *et al.*, 2016; Smith *et al.*, 2013). Here, we studied the effects of a single bolus of anti‐VEGFR2 on the ECM only 3 days after injection and in contrast showed that CAFs became activated and produced factors that, in fact, worked in favor of therapy delivery. We are the first to show that anti‐angiogenic therapy‐induced stromal activation results in improved chemotherapy delivery by degrading tumor HA content via hyaluronidase‐2. These effects could not be maintained after LT VEGFR2 inhibition in which stroma was no longer activated and HYAL2 production decreased, resulting in large amounts of HA in the tumors. These findings are in agreement with Rahbari and colleagues demonstrating increased presence of HA following prolonged bevacizumab treatment in colorectal liver metastases (Rahbari *et al.*, 2016). However, they propose that these effects are mediated by treatment‐induced tumor hypoxia while our experimental setup revealed that CAFs play a key role in this process. Zhao and colleagues reveal that inhibition of angiotensin II receptor type I with losartan normalizes the tumor stroma and improves drug delivery (Zhao *et al.*, 2019). Interestingly, Smith and colleagues describe that the specific architecture of the tumor stroma defines the tumor response to anti‐angiogenic therapies. Tumor cells surrounded by stroma that supports the majority of vessels (i.e., stromal vessel type) have a lower therapy response than tumors that have vessels distributed among the tumor cells (i.e., tumor vessel type) (Smith *et al.*, 2013). Our study shows that VEGFR2 targeting, in fact, induces a stromal vessel type, which indeed lowers the response of these vessels toward anti‐angiogenic treatment, and it would be interesting to determine whether similar effects follow other angiogenesis‐targeting agents.

Typically, the signaling cascades downstream of VEGFR2 lead to the activation of *NOS3* and the generation of NO (Hood *et al.*, 1998). Inhibition of the VEGFR2 signaling axis therefore results in decreased *NOS3* expression (Facemire *et al.*, 2009). This seems contradictory to our findings in which VEGFR2 inhibition resulted in an enhanced transcription of *NOS3*. However, we found that this activation is surprisingly mediated via the fibroblast growth factor receptor (FGFR). ST anti‐VEGFR2‐induced stromal activation results in enhanced FGF2 production, which can activate *NOS3*. This is supported by previous studies showing FGF2 to be a potent inducer of *NOS3* (Mata‐Greenwood *et al.*, 2008) signaling and explains how enhanced stromal activation status can lead to increased vasodilation. Given the availability of multiple FDA‐approved NO‐donating drugs, such as nitroglycerine and sodium nitroprusside, which are used in clinic for decades, this could be a promising strategy to improve delivery of cytotoxic agents during prolonged anti‐angiogenic treatment (Bryan, 2015). Moreover, this study underscores that anti‐angiogenic therapy has a wide range of effects and stresses the urge to shift the focus from studying ECs alone, to studying multiple cell types comprising the ECM in order to better understand the effects of VEGFR2 inhibition (Maracle *et al.*, 2018).

## 5 Conclusions

Altogether, our results reveal that ST VEGFR2 inhibition drastically changes the tumor architecture within just 3 days through HA degradation and NO production leading to increased intratumoral chemotherapy delivery. These results can provide new leads, such as the use of an NO donor or recombinant hyaluronidase enzyme, for the improvement of prolonged anti‐angiogenic treatment in the clinic. Unfortunately, as it is challenging to obtain follow‐up biopsies from patients with advanced‐stage EAC, these findings could not be validated in a clinical cohort. As multi‐agent DCE‐MRI is able to detect the observed changes in the ECM and hemodynamic function, image‐guided therapy could be a promising strategy to validate these findings and monitor and improve treatment outcome when anti‐angiogenic agents are used for longer periods of time in combination with cytotoxic agents.

## Conflict of interest

HWML has acted as a consultant for Eli Lilly and Company, and Nordic Pharma Group/Taiho and has received unrestricted research grants from Amgen, Bayer Schering Pharma AG, BMS, Celgene, Eli Lilly and Company, GlaxoSmithKline Pharmaceuticals, MSD, Nordic Pharma Group, Philips, and Roche Pharmaceuticals. MFB has received research funding from Celgene. JWW has received research funding from Celgene and Novartis. None of these were involved in drafting of the manuscript. All other authors declare no conflict of interest.

## Author contributions

AS, RK, MFB, and HWML conceived and designed the study; AS, RK, IJ, MCS, MGJTBL, EAE, SJH, SWT, CXM, MS, JJGHMB, JPM, JWW, RAAM, GJS, MFB, and HWML contributed to generation, collection, assembly, analysis, and/or interpretation of data; AS, RK, MFB, and HWML drafted or revised the manuscript; AS, RK, IJ, MCS, MGJTBL, EAE, SJH, SWT, CXM, CJAP, MS, JJGHMB, JPM, JWW, RAAM, GJS, MFB, HWML approved the final version of the manuscript.

## Supporting information


**Fig. S1.** Workflow of mice receiving long and short‐term anti‐angiogenic treatment.

**Fig. S2. **Long‐term anti‐angiogenic therapy induces apoptosis while proliferation and apoptosis are unaffected by short‐term treatment.

**Fig. S3.** Activation of CAFs is not mediated through direct stromal VEGFR2 inhibition.

**Fig. S4.** Short‐term anti‐angiogenic treatment degrades proteoglycans and stromal activation is correlated with hyaluronidase‐2 expression.

**Table S1.** Details of antibodies, heat‐induced epitope retrieval (HIER) and dilutions used for immunohistochemistry.
**Table S2.** List of primer sequences used for qRT‐PCR.
**Table S3.** *FGF2* and *MMP2* are strongly correlated with *NOS3* expression in the TCGA dataset.
**Table S4.** *FGF2* and *MMP2* are strongly correlated with *NOS3* expression in the Barbour dataset.
