# Supplementary figures and images for: Rapid stromal remodeling by short‐term VEGFR2 inhibition increases chemotherapy delivery in esophagogastric adenocarcinoma
Source: Mol Oncol. 2020 Mar 3;14(4):704–20. doi: 10.1002/1878-0261.12599 (PMC7138404; doi:10.1002/1878-0261.12599)

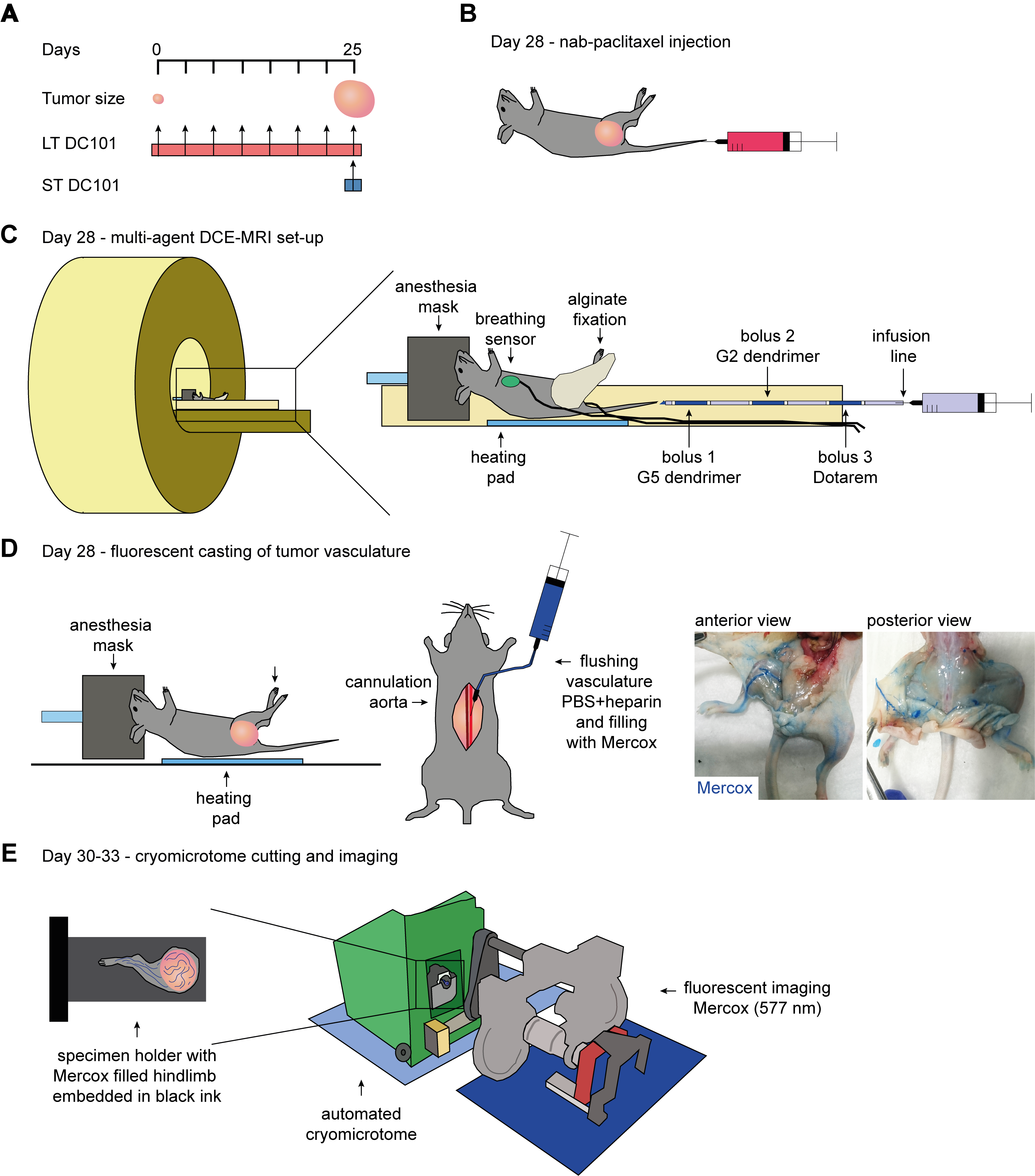

Supplement: Supplementary file 1 — Fig. S1. Workflow of mice receiving long and short‐term anti‐angiogenic treatment. [file MOL2-14-704-s001.tif]

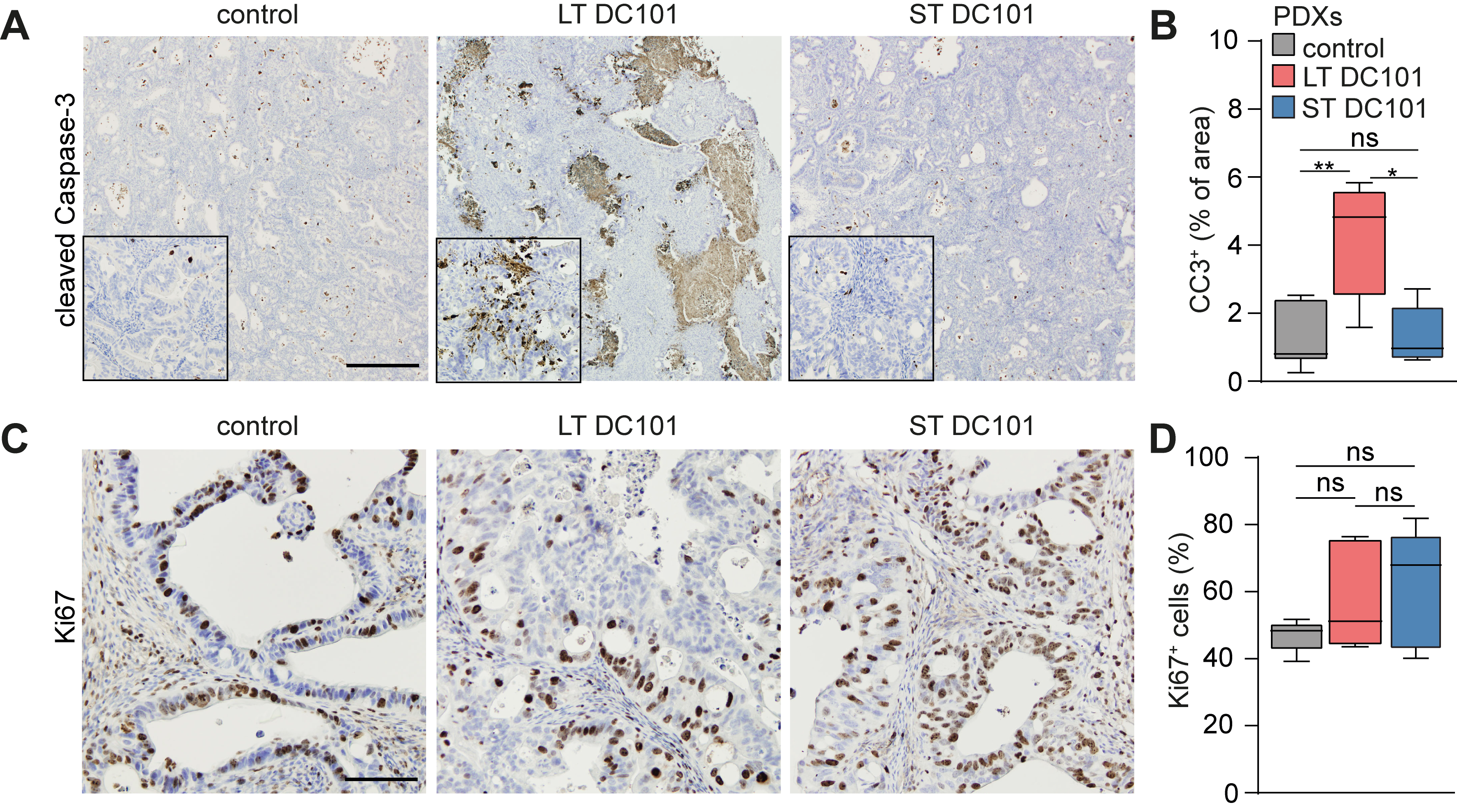

Supplement: Supplementary file 2 — Fig. S2. Long‐term anti‐angiogenic therapy induces apoptosis while proliferation and apoptosis are unaffected by short‐term treatment. [file MOL2-14-704-s002.tif]

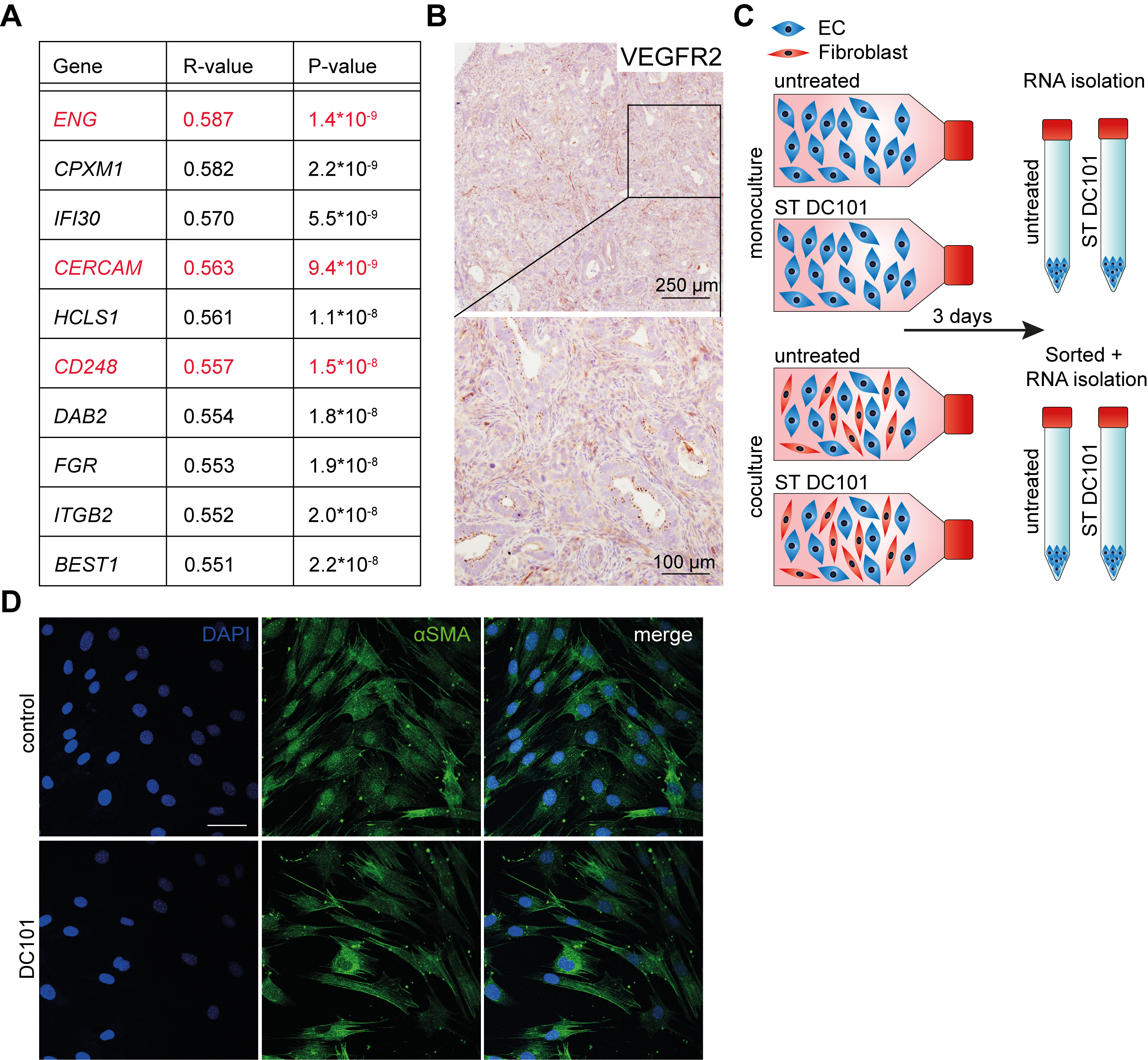

Supplement: Supplementary file 3 — Fig. S3. Activation of CAFs is not mediated through direct stromal VEGFR2 inhibition. [file MOL2-14-704-s003.tif]

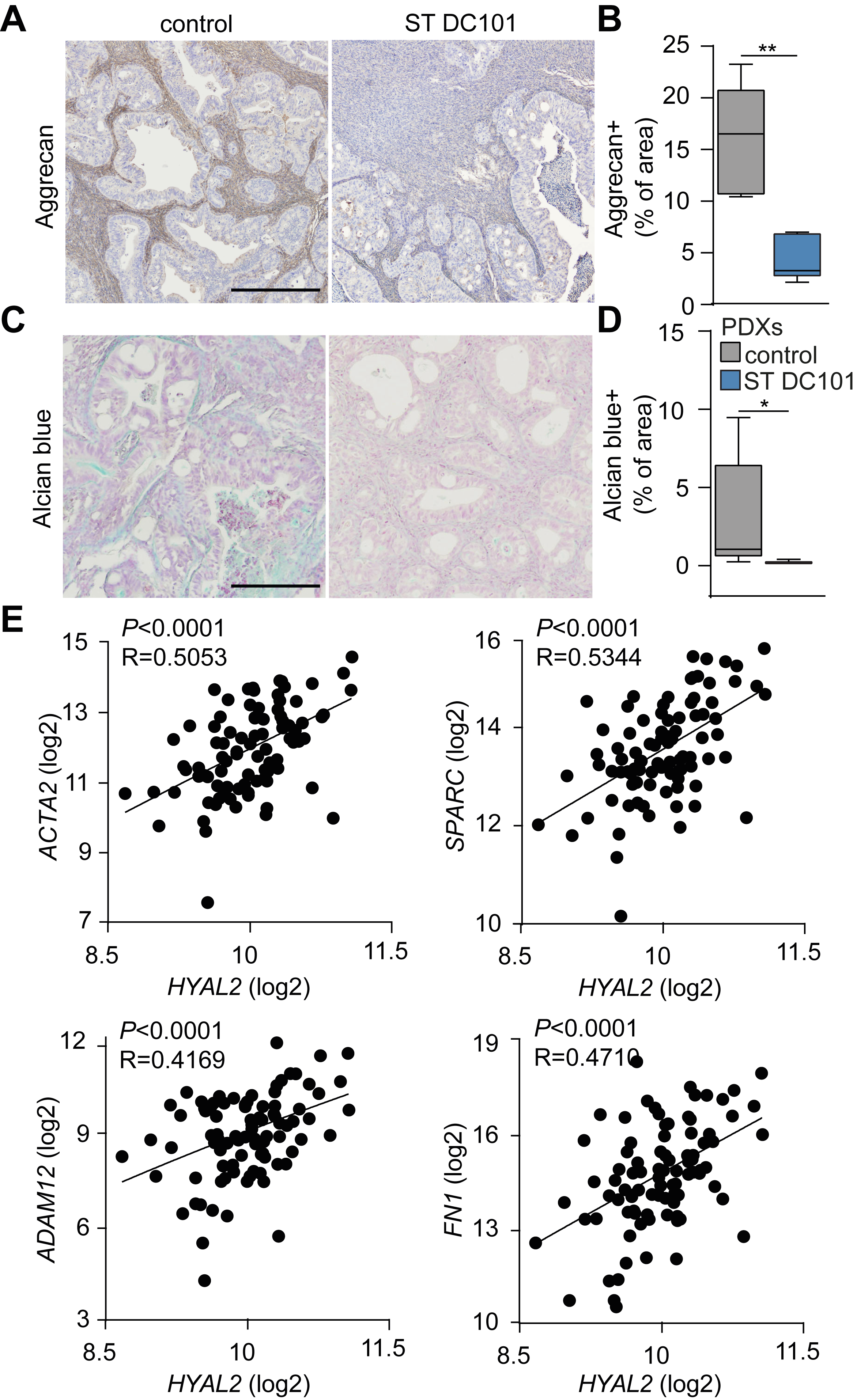

Supplement: Supplementary file 4 — Fig. S4. Short‐term anti‐angiogenic treatment degrades proteoglycans and stromal activation is correlated with hyaluronidase‐2 expression. [file MOL2-14-704-s004.tif]
