# Supplementary material for: Rapid stromal remodeling by short‐term VEGFR2 inhibition increases chemotherapy delivery in esophagogastric adenocarcinoma
Source: Mol Oncol. 2020 Mar 3;14(4):704–20. doi: 10.1002/1878-0261.12599 (PMC7138404; doi:10.1002/1878-0261.12599)
Supplement: Supplementary file 5 — Table S1. Details of antibodies, heat‐induced epitope retrieval (HIER) and dilutions used for immunohistochemistry. Table S2. List of primer sequences used for qRT‐PCR. Table S3. FGF2 and MMP2 are strongly correlated with NOS3 expression in the TCGA dataset. Table S4. FGF2 and MMP2 are strongly correlated with NOS3 expression in the Barbour dataset. [file MOL2-14-704-s005.docx]

**SUPPLEMENTARY INFORMATION**

**Rapid stromal remodeling by short-term VEGFR2 inhibition increases chemotherapy delivery in esophagogastric adenocarcinoma**

Anne Steins^1,2,12*^, Remy Klaassen^1,2*^, Igor Jacobs^3,4^, Matthias C. Schabel^5^, Monique G.J.T.B. van Lier^6^, Eva A. Ebbing^1^, Stefanie J. Hectors^7^, Sander W. Tas^8,9^, Chrissta X. Maracle^8, 9^, Cornelis J.A. Punt^2^, Maria Siebes^6^, Jacques J.G.H.M. Bergman^10^, Jan Paul Medema^1,12^, Johanna W. Wilmink^2^, Ron A.A. Mathot^11^, Gustav J. Strijkers^6^, Maarten F. Bijlsma^1,12§^ and Hanneke W. M. van Laarhoven^2§^

**SUPPLEMENTARY FIGURE LEGENDS**

**Figure S1. Workflow of mice receiving long and short-term anti-angiogenic treatment.** Treatment schedule of LT and ST DC101 treated tumors. Black arrows indicate the time points when DC101 (40 mg/kg) was administered i.p. (A) On day 28, three days after the last DC101 injection, mice were either intravenously injected with nab-paclitaxel (120 mg/kg) (B) or subjected to a multi-agent DCE-MRI scan (C). While under anesthesia, an infusion line containing 3 boluses with different sized contrasting agents was placed in the tail vein. Subsequently, the mouse was placed in a cradle on a heating pad with a breathing sensor and rectal temperature probe and the tumor-bearing hindlimb was embedded in alginate. After the multi-agent DCE-MRI scan, which took ~4 hours, the abdomen of the mouse was opened and the aorta was dissected and cannulated (D). The vasculature was flushed with PBS containing heparin until all was clear of blood. Subsequently, the vasculature was filled with Mercox under physiological pressure and left to polymerize. After filling of the vasculature, which took ~3 hours, the tumor-bearing hindlimb was fixated in PFA, embedded in black ink (E), and placed in a handcrafted automated cryomicrotome. After each cut section, a fluorescent image was made of the Mercox (577 nm) which would typically take a day to complete for the entire hindlimb.

**Figure S2. Long-term anti-angiogenic therapy induces apoptosis while proliferation and apoptosis are unaffected by short-term treatment.** (A) PDX tumors were stained for cleaved caspase-3 (CCS3) with IHC. Scale bar is 500 µm. (B) Quantification of CCS3 as percentage of area using ImageJ Software. n=10 for control group and n=5 for LT and ST DC101 groups. (C) PDX tumors were stained for Ki67 with IHC. Scale bar is 100 µm. (D) Quantification of Ki67 positive cells as percentage of DAB positive cells using ImageJ Software. n=7 for control group, n=6 for LT and n=5 for ST DC101 groups. **P*<0.05, ***P*<0.01. Error bars in all bar graphs represent SD. All Student’s *t*-test.

**Figure S3. Activation of CAFs is not mediated through direct stromal VEGFR2 inhibition.** (A) The TCGA dataset was used to identify the top 10 most significantly correlated genes to *NOS3*. Only EAC samples were selected, *P-* and R-value were determined by linear regression analysis. (B) PDX tumors were stained for VEGFR2 with IHC. Shown is a DC101 treated tumor. (C) Graphical representation of mono- and cocultures. Cerulean-labeled murine ECs were subjected to DC101 or PBS control for three days either in monoculture, or in coculture with mCherry-labeled murine CAFs. Subsequently, the cerulean positive ECs were cell sorted and RNA was isolated. (D) Murine CAFs were treated with DC101 or control for three days and stained for α-SMA using immunofluorescence. Scale bar is 50 µm.

**Figure S4. Short-term anti-angiogenic treatment degrades proteoglycans and stromal activation is correlated with hyaluronidase-2 expression.** (A) PDX tumors were stained for aggrecan with IHC. n=5 per group. Scale bar is 500 µm. (B) Aggrecan staining was quantified as percentage of area using ImageJ Software. Student’s *t*-test. (C) PDX tumors were histochemically stained for Alcian blue. n=5 per group. Scale bar is 250 µm. (D) Alcian blue staining was quantified as percentage of area using ImageJ Software. Student’s *t*-test. (E) Gene expression correlation between stromal activation markers *ACTA2, SPARC, ADAM12, FN1* and HA degrading enzyme *HYAL2* using the TCGA dataset selecting EAC samples only. *P*- and R-value were determined by linear regression analysis. **P*<0.05, ***P*<0.01. Error bars in all bar graphs represent SD.

**SUPPLEMENTARY TABLES**

| **Antibody** | **Heat-induced epitope retrieval** | **Dilution** |
| --- | --- | --- |
| Anti-CD31 (ab28364, Abcam) | Tris-EDTA buffer solution at pH 9, 20 minutes at 98ºC (Lab Vision™ PT Module™, Thermo Scientific, Waltham, Massachusetts, US) | 1:400 |
| Anti-Cleaved Caspase 3 (Asp175) (9661S, Cell Signalling) | Sodium citrate buffer solution at pH 6, 30 minutes at 95ºC (Lab Vision™ PT Module™) | 1:200 |
| Anti-Ki67 (SAB5500134, Merck) | Sodium citrate buffer solution at pH 6, 20 minutes at 98ºC | 1:2000 |
| Anti-alpha smooth muscle Actin (ab5694, Abcam) | Sodium citrate buffer solution at pH 6, 20 minutes at 98 ºC | 1:1000 |
| Anti-VEGFR2 (9698S, Cell Signaling Technology) | Tris-EDTA buffer solution at pH 9, 20 minutes at 98ºC | 1:500 |
| Anti-Hyaluronic Acid (ab53842, Abcam) | Pepsin (0.25% in 0.01M HCL) for 15 minutes at 37ºC (Sigma, Saint Louis, Missouri, US) | 1:100 |
| Anti-Aggrecan (AB1031, Merck Milipore) | Chondroitinase ABC (0.1 U/ml; in 50 mM Tris pH 8.0, 60 mM Sodium Acetate and 0.02% BSA) for 30 minutes at 37ºC (Sigma) | 1:500 |
| Rabbit anti-Sheep (6016-01, Southern Biotech) | NA | 1:3000 |

**Table S1. Details of antibodies, heat-induced epitope retrieval (HIER) and dilutions used for immunohistochemistry.**

**Table S2. List of primer sequences used for qRT-PCR.**

| Gene | Primer (5’🡪3’) |
| --- | --- |
| *hB2M* | \| Fwd: GTCTTTCAGCAAGGACTGGTC \| \| --- \| \| Rev: CTTCAAACCTCCATGATGC \| |
| *mB2m* | \| Fwd: CTTCAGTCGTCAGCATGG \| \| --- \| \| Rev: GTTCTTCAGCATTTGGATTTC \| |
| *mCol1a* | \| Fwd: TAGGCCATTGTGTATGCAGC \| \| --- \| \| Rev: ACATGTTCAGCTTTGTGGACC \| |
| *mActa2* | \| Fwd: CTGACAGAGGCACCACTGAA \| \| --- \| \| Rev: AGAGGCATAGAGGGACAGCA \| |
| *mSparc* | \| Fwd: CCAGGCAAAGGAGAAAGAAG \| \| --- \| \| Rev: TTCAGACCGCCAGAACTCTT \| |
| *mAdam12* | \| Fwd: GCTTTGGAGGAAGCACAGAC \| \| --- \| \| Rev: CGCATCAACGTCTTCCTTTT \| |
| *mFn1* | \| Fwd: TTCAAGTGTGATCCCCATGAAG \| \| --- \| \| Rev: CAGGTCTACGGCAGTTGTCA \| |
| *mMmp2* | \| Fwd: ACAAGTGGTCCGCGTAAAGT \| \| --- \| \| Rev: GTAAACAAGGCTTCATGGGGG \| |
| *mMmp10* | \| Fwd: CACCCTCAGGGACCAACTTA \| \| --- \| \| Rev: TGGAAGTTAGCTGGGCTTGT \| |
| *mMmp9* | \| Fwd: CAGCCGACTTTTGTGGTCTTC \| \| --- \| \| Rev: ATAGCGGTACAAGTATGCCTCTGC \| |
| *mHyal1* | \| Fwd: CTACCGTGGATGGAGAGGAGGA \| \| --- \| \| Rev: GTCTCCAGTCCTCGTGGGTT \| |
| *mHyal2* | \| Fwd: TGGAGACCTGCCAATACCTCA \| \| --- \| \| Rev: GCACTGGGTCCAACTGCAAT \| |
| *mHas2* | \| Fwd: GAAACTTCCTTCCACGACCCT \| \| --- \| \| Rev: TCACAATGCATCTTGTTCAGCTC \| |
| *mHas3* | \| Fwd: GTGGGCACCAGTCTGTTTG \| \| --- \| \| Rev: CCACTGAACGCGACCTCTG \| |
| *mNos3* | \| Fwd: TCAGCCATCACAGTGTTCCC \| \| --- \| \| Rev: ATAGCCCGCATAGCGTATCAG \| |
| *mFgf2* | \| Fwd: CGACCCACACGTCAAACTAC \| \| --- \| \| Rev: GCCGTCCATCTTCCTTCATA \| |

**Table S3.** ***FGF2* and *MMP2* are strongly correlated with *NOS3* expression in the TCGA dataset.** The TCGA dataset was used to identify significantly correlated genes to *NOS3* with gene category ‘drug target’ and KEGG pathway ‘pathways in cancer’. Overlapping genes between the TCGA and Barbour dataset are highlighted in yellow.

| **Gene** | **R-value** | **P-value** |
| --- | --- | --- |
| *PDGFRB* | 0.532 | 8.0*10^-8^ |
| *MMP2* | 0.532 | 8.2*10^-8^ |
| *CSF1R* | 0.531 | 8.6*10^-8^ |
| *EDNRA* | 0.430 | 2.6*10^-5^ |
| *AR* | 0.379 | 2.5*10^-4^ |
| *TGFB1* | 0.374 | 3.0*10^-4^ |
| *CSF2RA* | 0.371 | 3.4*10^-4^ |
| *FGF1* | 0.346 | 9.0*10^-4^ |
| *RARA* | 0.342 | 1.0*10^-3^ |
| *FGFR1* | 0.327 | 1.8*10^-3^ |
| *F2R* | 0.315 | 2.6*10^-3^ |
| *GNG2* | 0.311 | 3.0*10^-3^ |
| *ITGA2B* | 0.300 | 4.2*10^-3^ |
| *ABL1* | 0.290 | 5.9*10^-3^ |
| *PDGFRA* | 0.285 | 6.8*10^-3^ |
| *BCL2* | 0.283 | 7.2*10^-3^ |
| *FGF2* | 0.282 | 7.4*10^-3^ |
| *PTGER1* | 0.278 | 8.4*10^-3^ |

**Table S4. *FGF2* and *MMP2* are strongly correlated with *NOS3* expression in the Barbour dataset.**  The Barbour dataset was used to identify significantly correlated genes to *NOS3* with gene category ‘drug target’ and KEGG pathway ‘pathways in cancer’. Overlapping genes between the TCGA and Barbour dataset are highlighted in yellow.

| **Gene** | **R-value** | **P-value** |
| --- | --- | --- |
| *EDNRB* | 0.452 | 1.3*10^-3^ |
| *MMP1* | 0.442 | 1.7*10^-3^ |
| *MMP2* | 0.435 | 2.0*10^-3^ |
| *PDGFRB* | 0.416 | 3.2*10^-3^ |
| *FGF2* | 0.401 | 4.7*10^-3^ |
| *PTGS2* | 0.378 | 8.1*10^-3^ |
